# Virtual home-based palliative care during COVID-19: A qualitative exploration of the patient, caregiver, and healthcare provider experience

**DOI:** 10.1177/02692163221116251

**Published:** 2022-10

**Authors:** Daniel Vincent, Cayden Peixoto, Kieran L Quinn, Kwadwo Kyeremanteng, Genevieve Lalumiere, Allison M Kurahashi, Nathalie Gilbert, Sarina R Isenberg

**Affiliations:** 1 Division of Palliative Care, Department of Medicine, University of Ottawa, Ottawa, ON, Canada; 2 Clinical Epidemiology Program, Ottawa Hospital Research Institute (OHRI), Ottawa, ON, Canada; 3 Institut du Savoir Montfort, Ottawa, ON, Canada; 4 Department of Medicine, University of Toronto, Toronto, ON, Canada; 5 Temmy Latner Centre for Palliative Care, Sinai Health System, Toronto, ON, Canada; 6 Division of Critical Care, University of Ottawa, Ottawa, ON, Canada; 7 Regional Palliative Consultation Team, Elizabeth Bruyère Hospital, Ottawa, ON, Canada; 8 Home and Community Care Support Services Champlain, Ottawa, ON, Canada; 9 Bruyère Research Institute, Ottawa, ON, Canada; 10 Department of Medicine, University of Ottawa, Ottawa, ON, Canada; 11 Department of Family and Community Medicine, University of Toronto, Toronto, ON, Canada

**Keywords:** Telemedicine, virtual palliative care, COVID-19, palliative care, qualitative research

## Abstract

**Background:**

Due to the COVID-19 pandemic, many community palliative healthcare providers shifted from providing care in a patient’s home to providing almost exclusively virtual palliative care, or a combination of in-person and virtual care. Research on virtual palliative care is thus needed to provide evidence-based recommendations aiming to enhance the delivery of palliative care during and beyond the pandemic.

**Aim:**

To explore the experiences and perceptions of community palliative care providers, patients and caregivers who delivered or received virtual palliative care as a component of home-based palliative care during the COVID-19 pandemic.

**Design:**

Qualitative study using phone and video-based semi-structured interviews. Data were analyzed using thematic analysis.

**Setting/participants:**

A total of 37 participants, including community palliative care patients/caregivers (*n* = 19) and healthcare providers (*n* = 18) recruited from sites in Ottawa and Toronto, Ontario, Canada.

**Results:**

Overall, participants preferred in-person palliative care compared to virtual care, but suggested virtual care could be a useful supplement to in-person care. The findings are presented in three main themes: (1) Impact of COVID-19 pandemic on community palliative care services; (2) Factors influencing transition from exclusively virtual model of care back to a blended model of care; and (3) Recommended uses and implementation of virtual palliative care

**Conclusions:**

Incorporating virtual palliative care into healthcare provider practice models (blended care models) may be the ideal model of care and standard practice moving forward beyond the COVID-19 pandemic, which has important implications toward organization and delivery of community palliative care services and funding of healthcare providers.

**What is already known about the topic?**

- Because of the COVID-19 pandemic, many community palliative care providers shifted from providing in-person support in a patient’s home, to providing almost exclusively virtual palliative care or a combination of in-person and virtual care (phone or video).
- Despite increased use of virtual care, perceptions of the usefulness of virtual palliative care remain variable.

**What this paper adds?**

- This study describes the role and usefulness of virtual palliative care in the provision of community palliative care services and proposes the ideal model of community palliative care service provision, incorporating virtual palliative care services.
- A blended model of care (combination of in-person care and virtual palliative care) was perceived as being the ideal model of care, providing several advantages while still being able to deliver high quality palliative care.

**Implications for practice, theory or policy**

- Incorporating virtual palliative care into community palliative care service provision may be the ideal model of care and may become a new standard of practice beyond the COVID-19 pandemic. This has important implications toward organization and delivery of community palliative care services and funding of healthcare providers providing virtual palliative care.

## Introduction

The COVID-19 pandemic in Canada negatively impacted access to community palliative care services and exposed gaps through increased demand and greater strain on the palliative care workforce.^1,2^ Community palliative care services in Ontario, Canada is publicly funded and provided through Home and Community Care Support Services by community healthcare service providers, including physicians and nurse practitioners, nurses, social workers, spiritual care providers, physio- and occupational therapists, and personal support workers. Equipping healthcare service providers with digital technology to conduct home-based virtual palliative care was an immediate strategic response and an opportunity to enhance the delivery of palliative care during COVID-19.^3^ In Canada prior to the COVID-19 pandemic, most community palliative care healthcare providers provided almost exclusively in-person care but shifted to exclusively virtual palliative care or a combination of in-person and virtual care (phone or video) during the pandemic.^4,5^ Provision of virtual palliative care in the community is defined as a patient assessment or evaluation completed by a healthcare provider (physician, nurse, coordinator or other team member) by telephone or any videoconferencing platform. These assessments can include comprehensive consultations, reassessments following a change in clinical status, routine follow-up for symptom and medication management, and support for people and caregivers at the very end of life.

In response to the pandemic, in March 2020, healthcare providers developed necessary skills and capacity to provide virtual palliative care despite limited evidence to support its use and ideal delivery, as well as limited healthcare provider education on virtual care delivery.^6–9^ This transition added to the already-significant burden imposed on palliative homecare providers during the pandemic,^9^ which led to delays in the provision of palliative care and patient-provider communication.^10,11^

Despite increased use of virtual healthcare services, perceptions of the usefulness of virtual palliative care remain variable. Patients, caregivers and healthcare providers prefer in-person visits.^10,12,13^ Previous studies evaluating healthcare provider attitudes to virtual palliative care found that concerns related to privacy, rapport building and technological malfunction are considered barriers to uptake.^14–16^ Among patients, barriers include perceived usefulness of virtual care,^17^ and limited experience using technology,^10,18^ especially among frail and elderly individuals.^19^ Although there is a growing body of research evaluating virtual palliative care,^20–22^ further study into the perceptions and experiences of both those delivering and receiving virtual palliative care during and beyond the pandemic, is needed.^23^

While virtual palliative care improves the efficiency of healthcare systems by reducing provider travel times, decreasing the burden on emergency services, and lowering the cost of care per patient,^12,14,24–26^ to our knowledge, there is limited research exploring the role and usefulness of virtual palliative care in the provision of community palliative care services and the ideal model of care incorporating virtual palliative care from the perspectives of providers, patients and caregivers.

This multi-site, qualitative study aimed to explore the experiences and perceptions of community palliative care patients, caregivers, and healthcare providers who received/delivered virtual palliative care as a component of home-based palliative care during the COVID-19 pandemic in Ontario, Canada. In understanding the experiences of these palliative care patients, caregivers and providers, the study provides data to inform future policies that support home-based virtual palliative care.

## Methods

### Design

We used a qualitative study design. This multi-site study utilized a post-positivist lens, which is a pluralistic approach combining positivism and interpretivism, which deems there is no universal truth and purports that multi-dimensional evidence can be inferred by perceived data such as that in qualitative interviews.^27^ This study is reported following the Consolidated Criteria for Reporting Qualitative Research (COREQ) guidelines.^28^

1. Population:

Inclusion criteria for patients and/or their caregivers:

Patient or caregiver of a patient who received home-based palliative care through either the Regional Palliative Care Consultation Team or the Orleans Palliative Care Team in Ottawa or the Temmy Latner Centre for Palliative Care in Toronto, between mid-March 2020 and July 2021. Participant had to be at least 18 years of age, have participated in at least 1 virtual visit and be English or French speaking.

Inclusion criteria for palliative healthcare providers:

Healthcare provider who provided palliative care at home. Included providers could be community palliative care physicians, nurse practitioners or registered nurses who provided care in a most-responsible provider or palliative care consultant role, as well as Home and Community Care Support Services Champlain palliative care coordinators who provided home-based palliative care services to patients and their caregivers between mid-March 2020 and July 2021. In addition, providers needed to have participated in the delivery of at least 1 virtual encounter and were English or French speaking.

2. Sampling:

We used purposive sampling, which involves selecting participants according to the needs of the study.^29^

Healthcare providers purposely contacted community palliative care colleagues who had provided virtual care as well as patients and or caregivers who had previously received virtual palliative care for participation in the study. We aimed to achieve thematic saturation across both study sites in Ottawa and Toronto. Correspondingly, we employed constant comparison analysis^30^ to determine thematic saturation of the data.^31^ After 15 interviews conducted with patients/caregivers, and 16 conducted with healthcare providers, we did not identify any new themes and we deemed that thematic saturation was achieved. Data collection and analysis continued for two more caregiver and healthcare provider participants (total participants 37) to confirm that no new themes would be identified.

3 Recruitment:

Community palliative care patients and/or their caregivers were identified through chart review of the electronic medical records of multiple community palliative care teams including the Regional Palliative Consult Team and Orleans Palliative Care Team in Ottawa and the Temmy Latner Centre for Palliative Care in Toronto. Patients or caregivers identified through chart review were either directly recruited by a member of the research team who was also a healthcare provider with the respective practice, or indirectly recruited through a mailed recruitment letter.

A recruitment email was sent to all identified specialist community palliative healthcare providers in the Champlain region and the Temmy Latner Centre for Palliative Care, and those who responded to the email expressing interest were contacted by the study coordinator to schedule an interview.

All individuals who were eligible and expressed a desire to participate in the study were scheduled for a virtual study interview over phone or video via Zoom. Participants were provided a $25 gift card honorarium following participation.

### Data collection

We conducted semi-structured interviews between March 2021 and July 2021. We developed interview guides through discussions with community palliative care healthcare providers, qualitative researchers, and through conducting a literature review to explore salient concepts relevant to virtual palliative care. Our caregiver advisor also reviewed the patient/caregiver interview guide. Similarly, a community palliative healthcare provider reviewed the healthcare provider guide. See Appendices 1 and 2 for finalized interview guides.

Most interviews were conducted by the principal investigator (DV) and the study coordinator (CP). To prevent a conflict of interest, all participants identified from Orleans Palliative Care Team, where DV is a physician, were interviewed by one of the other investigators. Field notes were collected by completing an Interviewer Form (Appendix 3) immediately after each interview.

Prior to commencing the interview, the participants verbally completed a demographic survey: patient/caregiver (Appendix 4) and healthcare provider (Appendix 5).

With participant consent, interviews were audio recorded, and transcribed verbatim by an external company, Home Row Inc. All potentially identifiable information was redacted from the transcript. The one interview conducted in French was transcribed verbatim in French and translated into English for analysis by an official translator at the Montfort Hospital.

### Data analysis

This study conducted inductive thematic analysis, led by DV, CP, and SI, wherein data pertaining to the experiences of receiving/delivering virtual palliative care during the COVID-19 pandemic were coded and categorized into recurring themes.^32^ Analysis began with close readings of transcripts to gain a thorough understanding of the dataset. After reaching a common conceptualization of content, preliminary codes were arranged into a coding framework and inserted into MAXQDA 2020^33^ along with the interview transcripts.

DV and CP consensus coded eight transcripts (chosen at random) and met to discuss and share their codes until a consistent level of consensus was established. The coding framework was then adjusted based on shared meaning and coding frequency within the transcripts. Afterward, the remaining transcripts (*n* = 27) were split between DV and CP and individually coded. Following coding of all transcripts, DV and CP met to review all coded quotes to ensure consistency, and conduct thematic analysis to identify and define themes and subthemes in the data.

## Results

A total of 35 semi-structured interviews were conducted in this study. The mean duration of interviews was 31:58 min and ranged 19:05–58:49 min. Two (*n* = 2) interested primary caregivers were unable to participate due to not meeting inclusion criteria. The final study population included *n* = 37 participants recruited from both the Ottawa and Toronto sites (Table 1). Among patient/caregiver participants in this study, 5 were patients and 14 were caregivers. The mean age of patients/caregivers was 63.5 years (SD = 12.7) and more than half were female (11 of 19) (Table 2). Among provider participants, 13 were palliative care physicians, 2 were palliative care nurse practitioners, and 3 were Champlain palliative care coordinators. The mean provider age was 45.6 years (SD = 11.1), and the majority were female (14 of 18) (Table 3). The overarching list of themes, sub-themes and codes appear in Table 4.

**Table 1.** Overall recruitment by recruitment site and participant type.

Participant type	OPCT	RPCT	TLCPC	Total
Patient	2	3	0	5
Caregiver	4	2	8	14
Physician	5	0	8	13
Nurse Practitioner	0	2	0	2
Care Coordinator	0	3	0	3
Total	11	10	16	37

**Table 2.** Descriptive characteristics of community palliative care patient and caregiver participants (*n* = 19).

Demographic characteristic	*n*	%
Age	Mean = 63.5 years (SD = 12.7)	
Gender		
Female	11	57.9
Male	8	42.1
Highest level of education completed		
High school	5	26.3
Postsecondary certificate or diploma	3	15.8
Bachelor’s degree	8	42.1
Graduate degree (Masters or Doctorate)	3	15.8
Yearly household income		
<$20,000	0	0
$20,000−$40,000	4	21.0
$40,000–$60,000	1	5.3
$60,000−$80,000	3	15.8
>$80,000	11	57.9
Race		
Caucasian	18	94.7
East Asian	1	5.3
First language		
English	17	89.4
French	1	5.3
Other	1	5.3
Religious or spiritual affiliation		
Christianity	8	42.1
Judaism	3	15.8
No Affiliation	8	42.1
Primary medical diagnosis of patient resulting in palliative care		
Cancer	9	47.4
Cardiac disease	2	10.5
Respiratory disease	3	15.8
Neurological disease	4	21.0
Other	1	5.3
Relationship to patient*		
Spouse	6	42.9
Child	5	35.7
Sibling	2	14.3
Friend	1	7.1

* For Caregivers only; *n* = 14

**Table 3.** Descriptive characteristics of community palliative healthcare providers (*n* = 18).

Demographic characteristic	*n*	%
Age	Mean = 45.6 years (SD = 11.1)	
Gender		
Female	14	77.8
Male	4	22.2
Highest level of education completed		
Bachelor’s degree	3	16.7
Graduate degree (Masters or Doctorate)	2	11.1
Medical Doctorate (M.D.)	13	72.2
Race		
Caucasian	15	83.3
South Asian	2	11.1
East Asian	1	5.6
Primary language of practice		
English	17	94.4
French/Bilingual	1	5.6
Number of years working in healthcare	Mean = 18.9 years (SD = 11.6)	
Number of years working in current position	Mean = 7.9 years (SD = 6.4)	

**Table 4.** Organization of themes, sub-themes, and codes.

Theme	Subtheme	Code(s)
Theme 1: Community Palliative Care Services During the Pandemic	(1) Models of community VPC provision during the pandemic	Blended (coordinating)
		Blended providing
		Exclusively in-person
		Exclusively VPC
Theme 2: Factors Influencing Transition from Exclusively VPC Model of Care back to a Blended Model of In-person Care and VPC During the Pandemic	(1) Financial and non-financial factors influencing HCP decision to transition back to in-person care	HCP financial impact of VPC
		Patient request for in-person visit
		Availability of PPE
		Patient Diagnosis and condition
		Patient proximity to end of life
		Risk Tolerance
		Vaccination Status
		Personal life circumstances
	(2) HCP dissatisfaction with providing exclusively VPC	Decreased job satisfaction
		Increased job satisfaction
	(3) Limited ability to complete comprehensive assessments	Limited ability to conduct comprehensive assessments
		Follow-up Assessments
	(4) Rapport/relationship between HCP and patients/families through VPC	Lack of personal connection or breakdowns in communication
Theme 3: Recommended Uses/Policy Implications of VPC	(1) Ideal model of community palliative care service provision	Ideal model of care
	(2) Acceptance/satisfaction of VPC	Patient or caregiver satisfaction with VPC
	(3) Usefulness of VPC	Efficiency of care

Three predominant themes were identified: (1) Impact of COVID-19 pandemic on community palliative care services; (2) Factors influencing transition from an almost exclusively virtual palliative care model of care back to a blended model of care; and (3) Recommended uses and implementation of virtual palliative care

### Impact of COVID-19 pandemic on community palliative care services

The majority of providers described a sudden shift to near-exclusive virtual palliative care at the beginning of the pandemic as per public health COVID-19 prevention guidelines, including a recommendation that healthcare providers shift to providing virtual care where possible.

*“So, initially, when there was a lot unknown about transmission, most things were done virtual unless there was a reason to be in-person. So, the default then became virtual visits.” –* HCP17 (Care Coordinator)

*“So, in the very beginning, it was the majority of patients who were virtual care. . .we would do 80 or 90% virtual care at the time, and then the other 10 to 20% was in-person”* – HCP6 (MD/NP)

In addition, physician participants reported a preference to coordinate their virtual visits while the visiting nurse was present (in-person) in the patient’s home since this enabled a higher quality patient assessment. Community palliative care nurses in the home performed focused physical exams and provided this information to physicians by phone or through a video platform.

*“I really have found that it works best when somebody else is there other than the family and the patient. I think being able to coordinate the virtual visit with somebody who’s in the home makes a big difference.” –* HCP1 (MD/NP)

*“For many of our patients, they might not have an email, they might not have a device. So, sometimes, it was just easier to FaceTime really briefly with the nurse for the physical assessment part of it. And I’d be like, show me this, show me their cath. . .then I would call them back and finish the rest over the phone”* – HCP4 (MD/NP)

These hybrid visits were facilitated by video technology and collaboration and coordination between physicians and nurses.

### Factors influencing transition from an almost exclusive virtual model of care back to a blended model of care

As the pandemic progressed, providers moved to a more blended care model.

*“I would say that over time, [we’ve] shifted to providing the majority of care in person and maybe 20% of that is virtual”* – HCP6 (MD/NP)

Providers described several factors influencing change from an almost exclusively virtual model of care at the beginning of the pandemic to a blended model of care. This theme includes four subthemes:

(1) Financial, COVID-19-related, and patient factors influencing provider decision to transition back to in-person care

Physicians in Ontario, Canada are renumerated through multiple possible funding models including alternative funding plans, salaried positions, or Ontario health insurance plan fee-for-service claim submission. Physicians billing fee-for-service described that Ontario palliative fee-for-service billing codes provided significantly less compensation for virtual palliative care than in-person care in the home, thus incentivizing in-person visits. This had a substantial financial impact and influenced the amount of virtual care provided by physicians who are paid through fee-for-service.

*“The fees are heavily skewed towards in-person care. . .you could be looking at 60% or 70% less income. You have to take that into account when you’re deciding on how you’re going to practice.”* – HCP1 (MD/NP)

*“When we were doing mostly virtual care in April and May, most of our pay were significantly reduced as a result of that. Because a lot of the home visits that we do, there’s premiums that we get for doing the in-person visits. So, yeah, it was a significant reduction for all of us, I think*” – HCP6 (MD/NP)

COVID-19-related factors that influenced provider decision to transition back to in-person care included improved availability of personal protective equipment, individual COVID-19 risk tolerance, being vaccinated against COVID-19, and provider personal life circumstances (e.g. having children attending school virtually).

*“I went back to full on providing in-person care, particularly as our PPE concerns went down. I’m vaccinated, which makes me a little more confident, at least for my self-protection. . .We all have kids, and so adjusting to what was happening at home and being able to manage the home life and the kids at home, but also trying to decrease our exposures” – HCP3 (MD/NP)*

In addition, providers and caregivers described several patient specific factors that led to in-person visits rather than virtual patient encounters despite the COVID-19 pandemic lockdown and recommendation that healthcare workers provide virtual care where possible. Factors included direct patient or caregiver request and patient diagnosis and condition, including proximity to end of life.

*“The palliative care doctor, he would come in whenever I called him or requested him to come in. . .I appreciated the in-person visit whenever I could get it because it was the scariest time in my life” – CG13*

*“If [patients] were more stable, I was certainly more comfortable seeing them virtually. . . I think as much as actively dying patients could be very easily managed by phone a lot of the time or virtually a lot of the time, I do find I do a lot of those visits in person because just to be able to provide that sort of support to the family” – HCP12 (MD/NP)*

Providers and patients/caregivers both expressed the importance of patient factors contributing to more in-person care despite the COVID-19 pandemic.

(2) Provider satisfaction with delivering virtual palliative care

Most healthcare providers described feeling unsatisfied providing exclusively virtual care at the beginning of the pandemic but were satisfied with having the ability to use virtual care as a means/tool in providing community palliative care services to patients and families in a blended model of care (in-person and virtual care).

*“The time when we were doing heavy virtual care wasn’t necessarily for us, but it’s also nice knowing that it is a tool that’s there if we need it and if it’s appropriate for the patient.”* – HCP6 (MD/NP)

Providers liked having the freedom to use virtual palliative care at their discretion pending the circumstances of the patient, but did not like when virtual care was used almost exclusively.

*“My satisfaction with palliative care done 100% virtually was at an all time low. It was just, I missed being in people’s homes and having that connection. I would say virtual care as an addition to in-person visits is probably a net positive”* – HCP4 (MD/NP)

(3) Limited ability to complete a comprehensive assessment through virtual palliative care

Providers described being uncomfortable and dissatisfied providing exclusively virtual care to patients and their families since they were not able to conduct comprehensive patient assessments virtually and consequently felt they were not providing high quality care. In-person assessments allowed for more thorough evaluation of physical symptoms, functional needs and accessibility of the home environment, and even psychosocial needs.

*“You really need to see that patient in person to get the most accurate assessment, to accompany patients as they’re declining in terms of their functional status. And so, often, I felt that there was a lot of missing information”* – HCP18 (MD/NP)

*“When I was doing exclusively virtual care, I was not very satisfied with the provision. I did feel that I was missing out on cues that the patient was giving me or things that you would really only see on a physical exam or when you’re actually looking at them. Subtle things that would be really helpful information I felt I was missing”* – HCP2 (MD/NP)

Caregivers also described concerns regarding limitations of virtual care, namely the potential for incomplete medical assessments.

*“But my feeling about the virtual care is that . . .there’s a potential to miss the overall picture”*– CG3

Providers described multiple challenges engaging in goals of care discussions and clarifying a patient’s goals of care by phone or video as compared to an in-person assessment in the patient’s home.

*“I do like to see people in-person, especially initially, because I find it just gives me. . . I just feel like I’ve got a more comprehensive, plus I feel I establish a more direct relationship with them. . . And I find sometimes, it’s difficult to have, though I have attempted, more kind of serious goals of care kind of conversations. I have done virtually, but I find they’re more successful in-person.”* – HCP8 (MD/NP)

*“Because the one thing that’s super hard for virtual care is talking about goals of care. Right ?”* – HCP4 (MD/NP)

(4) Rapport/relationship between providers and patients/caregivers

Patients and caregivers perceived that virtual care negatively impacted the quality of palliative care they received since it impeded the ability to establish personal connections with providers. Similarly, providers in this study reported that virtual palliative care limited their ability to form personal connections with patients and their caregivers.

*“I understand why they’re being done by Zoom, and I find our nurse practitioner has been very good, but I still just sort of feel like if she was here, the connection might be a little different, just a little more personal.”*− P4&CG6

“*I did feel there was a lot lost there as well, in terms of forming a relationship with the family and . . . with the patient.”–* HCP2 (MD/NP)

### Recommended uses and implementation of virtual palliative care

Participants described multiple recommended uses and an ideal model of care incorporating virtual care. This theme includes three subthemes:

(1) Ideal model of community palliative care service provision

Providers, patients and caregivers perceived in-person visits in the home as most desirable, but noted important roles for virtual care in the provision of care. Participants described the ideal model of community palliative care service provision as a model incorporating in-person visits and virtual care (blended model).

*“The ideal model would be making home visits if indicated for complex clients, if you can’t resolve something on the phone. I think that would be the ideal model. The norm shouldn’t have to be, you make a home visit to see the client every time. Because as we’ve shown in the last year, we haven’t done that.” –* HCP7 (Care Coordinator)

*“We’re able to sort of pick and choose what we think is most appropriate for us and for our patients, in terms of the amount of in-person and virtual care that we provide. . . I think moving forward, that can sort of be decided by the person who’s practicing” –* HCP6 (MD/NP)

(2) Patient and caregiver acceptance and satisfaction of a blended model of care

In general, patients and caregivers did not feel that virtual care worsened the overall quality of palliative care they received at home as long as providers were available for an in-person visit when requested. Similarly, providers felt that patients and caregivers were satisfied with virtual palliative care services, as long as there continued to be a mixture of in-person assessments and virtual care.

*“Well, I think that expectation of having visits all the time puts a big strain on the doctors. The combination, I think, is a more reasonable approach. But knowing that they would come if I felt concerned, either the nurse or the doctor, they were both very cooperative.” –* CG9

*“If I only did virtual appointments, I think they may not be as satisfied. Because now, I do a sort of a combination of virtual and in-person, and I think that’s the key.”–* HCP8 (MD/NP)

(3) Perceived usefulness of virtual palliative care (efficiency)

Providers described a blended model as both useful and efficient, and reported being more accessible and able to take on greater palliative care patient caseloads with less travel by providing care virtually.

*“I think that it has been efficient, it’s reduced the amount of driving I’ve had to do. It saves time. I can do more virtual appointments than face-to-face, so that has been helpful.”* – HCP8 (MD/NP)

Patients and caregivers explained that virtual palliative care helped avoid unnecessary in-person visits in the home and described its benefits for uncomplicated problems or symptoms or for patients whose condition was stable.

*“If it was technical, dealing with levels of medications, if it was dealing with discussing test results, if it was anything like that, it makes sense to do a Zoom call.”* – P6

## Discussion

### Main findings/results of the study

The purpose of this multi-site, qualitative study was to explore the experiences and perceptions of community palliative care patients, caregivers, and healthcare providers who received or delivered virtual palliative care during the COVID-19 pandemic in Ontario, Canada. Three predominant themes were identified: (1) Impact of COVID-19 pandemic on community palliative care services; (2) Factors influencing transition from an almost exclusive virtual model of care back to a blended model of care; and (3) Recommended uses and implementation of virtual palliative care

Similar to previous research on virtual care, the current study found a preference for in-person visits among both patient/caregivers^10,12^ and providers.^33^ Providers developed strategies and tools to complete high quality virtual patient assessments but perceptions of care quality were highest when patients were seen in-person. In line with our post-positivist lens, we observed a multi-dimensional perception of virtual palliative care. For example, providers articulated that they were dissatisfied with delivery of virtual care exclusively; whereas, patients were more accepting of virtual care with the caveat that in-person care be available as appropriate.

Patients and caregivers also reported experiencing diminished rapport/relationships during the pandemic with their providers, and vice versa, due to the use of almost exclusively virtual care as a substitute for in-person care. These results are corroborated by recent literature,^20,21^ and emphasize the importance of providers being cognizant that virtual care may negatively affect patient rapport. However, according to Collier et al., video platforms allow community palliative care providers to relate and connect with patients and their caregivers in a way not possible by telephone.^15^

Our study found that most providers quickly shifted back to primarily in-person care, but continued to provide virtual palliative care during the pandemic when it was felt to be appropriate. Providers described several COVID-19 related factors which contributed to the transition to a blended model of care, most importantly, the availability of PPE, COVID-19 vaccination and provider and patient preference for in-person visits. To our knowledge, these are novel findings in the palliative care literature. Furthermore, physicians renumerated by fee-for-service in this study reported a significant loss of income due to Ontario’s fee-for-service billing codes for virtual palliative care, which influenced the amount of virtual care they provided. Therefore, policy interventions should direct fee codes toward incentivizing virtual palliative care for fee-for-service physicians, while balancing the ongoing need for in-person care.

In corroboration with previous research,^13,34^ our study showed that virtual palliative care was not found to be a suitable replacement for in-person care, which was perceived by participants to be the gold standard of community palliative care. However, participants described advantages of a blended community care model, incorporating virtual palliative care, including avoiding “unnecessary” in-person visits in the home, greater accessibility by phone or video to palliative care providers and reduced patient and caregiver COVID-19 exposure risk due to reduced frequency of healthcare providers in the home. Moreover, providers in our study reported virtual care facilitating more efficient care, that is, being able to take on greater palliative care patient caseloads, or complete more visits with patients and their caregivers. These findings are supported by Salem et al., who found that remotely supporting patients via telemedicine provides them with timely access to palliative care, while allowing providers to be more efficient in care provision.^12^

Participants proposed an ideal model of community palliative care provision beyond the pandemic as one that incorporates both in-person visits and virtual care. These results are supported by Jess et al., who found that providers, patients and caregivers consider the incorporation of virtual palliative care as positive, and that its use may have the potential to reduce overall healthcare expenditures.^13^ Given our findings, we suggest providers consider a blended community palliative care model, tailoring the proportion of in-person visits and virtual care through shared decision making with patients and their caregivers. Local policy interventions will be needed to set practice standards for the use of virtual palliative care.

### What this study adds

This study addresses gaps in the literature by exploring the role and usefulness of virtual palliative care and the ideal model of community palliative care provision incorporating virtual care. Participants described both positive and negative impacts of delivering or receiving virtual palliative care on their perception of the quality of palliative care. Participants reported satisfaction with virtual care as long as there continued to be some in-person assessments when required. Our findings suggest that providers should transition to a blended model of care (in-person and virtual care) moving forward beyond the COVID-19 pandemic. This was perceived by participants as the ideal model of care. However, further research is needed to explore the role of virtual palliative care in community palliative care service provision models.

### Strengths and limitations

Our study sample included fewer patients (*n* = 5) compared to caregivers (*n* = 14) due to difficulty in recruiting patients who were still living. In addition, of the 18 providers in our sample, most were physicians (*n* = 13) due to a limited number of available nurse practitioners and difficulty recruiting palliative care coordinators. Therefore, study results may not necessarily be representative of those groups. In addition, visiting community palliative care nurses, who were largely unable to shift to virtual care during the pandemic, were not included and study results may not reflect their perceptions and experiences.

Our patient/caregiver cohort lacked diversity regarding language, ethnicity, and income level, in addition to the majority being highly educated (Table 2). Moreover, our provider cohort was largely Caucasian and English speaking (Table 3). Therefore, we cannot conclude if our results are generalizable to minority or non-English speaking community palliative care patients, caregivers and healthcare providers. However, the researchers interviewed all individuals who expressed interest in the study and were eligible to participate to help prevent bias in sampling. Regardless, further research investigating perceptions and experiences of receiving and delivering virtual palliative care during COVID-19 among an ethnically diverse sample of patients/caregivers and providers is needed to confirm the generalizability of our findings.

Our study is strengthened by the use of purposive sampling across multiple study sites, which increased the variety of patient, caregiver and provider perspectives/experiences in our sample. Moreover, this study conducted during the COVID-19 pandemic uniquely captured the experiences and perspectives of healthcare providers, as well as patients and or caregivers.

## Conclusion

The pandemic provided a timely opportunity to explore the perspectives of providers, patients and caregivers in providing and receiving virtual palliative care to identify the usefulness of virtual care in the provision of community palliative care services. Our findings suggest that a blended model of care, may be the ideal model of this care beyond the pandemic, providing several advantages while allowing the delivery of high-quality palliative care. Virtual care will continue to have a role in community palliative care provision, but it may not always an effective replacement for in-person patient assessments. Our study helps find the optimal balance between the use of in-person and virtual visits, and has important implications toward the organization and delivery of community palliative care services. Health policy interventions will be needed to set standards for the use of virtual palliative care and take advantage of incentive levers to ensure it is used properly.

## Appendix 1 – Patient and caregiver interview guide

### Patient/Caregiver Interview Guide

#### Context of care

1. Before we begin the interview, can you tell us what palliative care means to you? (ie. participants own understanding of what palliative care is)
2. What were your expectations, hopes, concerns with receiving home-based palliative care?
3. Please describe your roles and experience with palliative care (e.g. patient, caregiver, duration of care, etc.)
  - If caregiver, what are your responsibilities in relation to providing care for the patient?
4. Did you receive home-based palliative care prior to the pandemic? (i.e. before March 2020)
  - If yes, proceed to questions 5
  - If no, proceed to question 7
5. What was your experience of home-based palliative care prior to the COVID-19 pandemic?
  - Probe: How would you typically see/receive your palliative care services?
  - Probe: Did you receive any virtual palliative services (telephone, email, video calls, etc.) at home prior to the pandemic?

### Impact of COVID-19

6. Have your community/home-based palliative care services *changed* since the start of the pandemic? If yes, how so?
  - Probe: How did these changes impact your relationship with your palliative care team?
7. How did the pandemic impact the care you received from your community palliative care team?
  - Probes: did you feel the need to cancel any in-person visits or services?
  - Did your palliative care provider(s) ever express concern or reservations about entering your home? If yes, do you feel it impacted the care you received or your relationship with your palliative care team?

### Virtual palliative care

8. Can you tell us what virtual healthcare means to you? (i.e. participant’s own understanding what of virtual health care is)?
9. Prior to receiving virtual palliative care, what were your expectations, hopes or concerns with receiving palliative care virtually? (e.g. increase safety/convenience, privacy concerns, apprehensive with unfamiliar technology, quality of care)
  - Probe: Were those expectations/hopes/concerns realized? If so, how were they addressed?
10. Describe your experience receiving virtual palliative care in the home
  - Probes: How were you/your loved one cared for virtually by their palliative care team?
    - What did the doctors/nurse ask during the phone calls/virtual visits?
    - Can you provide an example of a virtual visit you had where the doctor/nurse did not end up visiting you in-person after?
    - Can you provide an example of a virtual visit you had where the doctor/nurse followed up with you in-person shortly after?
  - What platform did you use to connect? (e.g. OTN, facetime, zoom)
    - If not video, why not?
  - What did you like, what did you not like?
  - If patient: Did the use of virtual care impact your caregiver’s participation in the visit?
  - If caregiver: Did the use of virtual care impact your participation in the visit?
11. Do you feel that using virtual palliative care impacted the quality of care you received?
  - Probes: why or why not? How so?
12. What were some of the biggest challenges associated with receiving virtual palliative care? (e.g. internet connection or other technological issues, difficulty finding an appropriate place within the home for the call, etc.)
  - Probes: Where these challenges addressed? Why or why not? (e.g. appropriate training, guidance from HCP)
  - Were you concerned about being able to connect to the virtual care platform (e.g. having fast internet connection, having an up-to-date device, being able to download the right apps, etc.)
  - Do you ever worry about privacy when discussing your/your loved one’s care with your provider using virtual palliative care?
13. In your opinion, how can virtual palliative care be improved?
14. Have you received non-palliative virtual care during the pandemic?
  - If yes, how did it compare to the virtual palliative care you received?
15. Having received virtual palliative care services, do you prefer continued in-person visits or the use of virtual palliative care services (telephone, email, video calls, etc.)?
  - Probe: would you still opt for in-person care? Why or why not?
  - Do you think virtual care should be part of the new “normal/standard” for receiving home-based palliative care?

If the participant is a bereaved caregiver, proceed to question 16. If not, skip to question 17.

16. Towards the end of your loved one’s life (when condition began to decline), did the use of virtual palliative care cause any specific challenges associated with receiving palliative care?
  - Probes: Were these challenges addressed? Why or why not?
  - Did you have any concerns about how the use of virtual palliative care during the COVID-19 pandemic might have impacted your loved one’s quality of death?
17. Is there anything else you would like to share about virtual palliative care?

## Appendix 2– Healthcare provider interview guide

### Healthcare Provider Interview Guide

#### Context of care

1. Please describe your current role within palliative care (e.g. physician/nurse practitioner, place of work, rural vs, urban)?
2. What was your experience delivering home-based palliative care prior to the COVID-19 pandemic?
  - Probe: how would you typically see/treat/communicate with patients?
3. What was your experience with virtual palliative care prior to the pandemic?
  - Probes: What virtual care methods or platforms did you use? (telephone, email, video calls, etc.)
  - How many times per week were you using virtual care?
  - What proportion of your patients did you use virtual care with?
  - How comfortable were you using virtual care?

#### Impact of COVID-19 Pandemic

4. How did the pandemic impact your teams’ delivery of home-based palliative care? (e.g. balancing personal safety with care for patient, PPE shortage, patients/caregivers refusing in-person care)
  - Probe: what strategies did your team take in response to these changes? (e.g. virtual care, blended model of care, joining virtually with those providing in-person care)
5. Can you specify if your use of virtual care during the pandemic was: only virtual care or a blended model of in-person and virtual care?
  - Probe: If you offered a blended model, how did you decide when to offer a patient/caregiver virtual versus in-person home visits?

#### Virtual care during COVID-19

6. What platform did you use to connect for virtual care? (e.g. OTN, FaceTime, Zoom)?
7. How did the use of virtual palliative care impact the care you provided?
  - Probes: How did the use of virtual care impact the relationship you had with your patients?
  - Probes: What sort of health concerns would you address over a virtual visit? What sort of concerns would prompt you to visit a patient in-person?
  - Probes: Did patients and or their caregivers prefer an in-person or virtual visit?
8. Have you received any training on providing virtual care?
  - Probes: What helped you in getting comfortable with using VPC?
  - Probes: How have you provided support to other colleagues regarding the use of VPC or vice versa?
9. How do you think your use of VPC impacts your care delivery for patients living in rural areas?
10. Do you think it is important to have a caregiver present during a virtual visit? Why/why not?
  - Probes: Do you think it is important to have a nurse present? Why/why not?
11. What were some of the major challenges you experienced with providing virtual palliative care to patients and their families during COVID-19? (e.g. technological difficulties, interruption of workflow, resistance from patients/caregivers, confidentiality issues, inability to physically assess patients)
  - How did you respond to these challenges?
  - How did the use of virtual palliative care affect interprofessional collaboration?
12. What were some of the major advantages of virtual care?
13. Are there any specific patient factors that you find make using virtual palliative care easier or more difficult?
14. Did virtual care impact access to community palliative care services for patients and their families?
  - How so?
15. Were certain patients unable to access care virtually?
  - If so, why?
16. How did virtual care impact your overall satisfaction in providing community palliative care services?
17. Given your current remuneration model, was there a financial impact of providing in-person versus virtual palliative care services to patients and their families?
18. How has your experience and perception of virtual palliative care changed during the pandemic? (e.g. comfort and confidence using virtual care)
19. Do you think virtual care should be part of the new “normal standard” for delivering home-based palliative care?
  - Why/why not?
  - Do you think healthcare providers’ participation in virtual care should be voluntary? Why or why not?
  - Do you think that training for virtual care should be mandatory for HCPs?
20. Is there anything else that I haven’t asked about that you would like to share?

## Appendix 3 – Interviewer form

### Interviewer data collection form

Interview Information

Date:

Interviewer Initials:

Participant ID:

Start Time:

End Time:

Length of Interview:

Interview Location (teleconference or video call):

Participant Type (healthcare provider – specify, patient, or caregiver):

Description (i.e. setting and participant):

Key Insights (i.e. poignant summary points):

Emerging Themes (i.e. patterns emerging across interviews):

Items to Flag Regarding Data Collection (i.e. for discussion at team meeting):

Self-Reflexive Notes (e.g. assumptions, lessons learned):

## Appendix 4 – Patient/caregiver demographic form

Patient and Caregiver Information Form

Participant ID: ____________

Date: ____________

**1. What is your age:** ____________
**2. What is your gender**: _______
  - Female
  - Intersex
  - Male
  - Trans – Female to Male
  - Trans – Male to Female
  - Other (please specify): ________
  - Prefer not to answer
  - Don’t know
**3. Education level**
  - 0–8 years of study
  - Some high school
  - High school graduate
  - Some postsecondary
  - Postsecondary certificate or diploma
  - Bachelor’s degree
  - Above bachelor’s degree
  - Prefer not to answer
**4. Level of yearly income**
  - <$20,000
  - $20,000–$40,000
  - $40,000–$60,000
  - $60,000–$80,000
  - $80,000
  - Prefer not to answer
**5. Which of the following best describes your racial or ethnic group? Check ONE only.**
  - Asian – East (e.g. Chinese, Japanese, Korean)
  - Asian – South (e.g. Indian, Pakistani, Sri Lankan)
  - Asian – South East (e.g. Malaysian, Filipino, Vietnamese)
  - Black – African (e.g. Ghanaian, Kenyan, Somali)
  - Black – Caribbean (e.g. Barbadian, Jamaican)
  - Black – North American (e.g. Canadian, American)
  - First Nations
  - Indian – Caribbean (e.g. Guyanese with origins in India)
  - Indigenous/Aboriginal – not included elsewhere
  - Inuit
  - Latin American (e.g. Argentinean, Chilean, Salvadoran)
  - Métis
  - Middle Eastern (e.g. Egyptian, Iranian, Lebanese)
  - White – European (e.g. English, Italian, Portuguese, Russian)
  - Mixed heritage (e.g. Black – African and White – North American) (Please specify): _________
  - Other(s) (Please specify): _________________
  - Prefer not to answer
  - Do not know
**6. What is your first language (Mother tongue)?**
  English
  French
  Other (Please specify): 
    ________________________________
  **7. What is your religious or spiritual affiliation? Check ONE only.**

**Table:**

• I do not have a religious or spiritual affiliation	• Native Spirituality
• Animism or Shamanism	• Pagan
• Atheism	• Protestant
• Baha’i Faith	• Rastafarianism
• Buddhism	• Roman Catholic
• Christian –*not included elsewhere*	• Sikhism
• Christian Orthodox	• Spiritual
• Confucianism	• Unitarianism
• Hinduism	• Zoroastrianism
• Jainism • Jehovah’s Witnesses	• Other (Please specify):
• Judaism	• Prefer not to answer
• Islam	• Do not know

8. Please indicate your relationship to the patient (i.e. answer this as though your response is “I am the patient’s [insert]”)
  - Aunt/uncle
  - Boyfriend/girlfriend
  - Child
  - Fiancé
  - Friend
  - Grandchild
  - Grandparent
  - Parent
  - Sibling
  - Spouse (married or common law)
  - Other
    If other, please specify the patient’s relationship to you:
**9. What medical diagnosis has led the patient to receive home-based palliative care?**
  - _______________________________
    - If cancer, please specify the type: ___________
  - Prefer not to answer
**10. Do you live in the same residence as the patient?**
  - Yes (if yes, whose residence did you live at: __________)
  - No

## Appendix 5 – Healthcare provider demographic form

Healthcare provider information form

Participant ID: ____________

Date: ____________

**1. What is your age:** ____________
**2. What is your gender**: _______
  - Female
  - Intersex
  - Male
  - Trans – Female to Male
  - Trans – Male to Female
  - Other (please specify): ________
  - Prefer not to answer
  - Don’t know
**3. Education level**
  - High school graduate
  - Postsecondary certificate or diploma
  - Bachelor’s degree
  - Master’s Degree
  - PhD
  - MD
  - Prefer not to answer
**4. Which of the following best describes your racial or ethnic group? Check ONE only.**
  - Asian – East (e.g. Chinese, Japanese, Korean)
  - Asian – South (e.g. Indian, Pakistani, Sri Lankan)
  - Asian – South East (e.g. Malaysian, Filipino, Vietnamese)
  - Black – African (e.g. Ghanaian, Kenyan, Somali)
  - Black – Caribbean (e.g. Barbadian, Jamaican)
  - Black – North American (e.g. Canadian, American)
  - First Nations
  - Indian – Caribbean (e.g. Guyanese with origins in India)
  - Indigenous/Aboriginal – *not included elsewhere*
  - Inuit
  - Latin American (e.g. Argentinean, Chilean, Salvadoran)
  - Métis
  - Middle Eastern (e.g. Egyptian, Iranian, Lebanese)
  - White – European (e.g. English, Italian, Portuguese, Russian)
  - Mixed heritage (e.g. Black – African and White – North American) (Please specify): ______________
  - Other(s) (Please specify): _________________
  - Prefer not to answer
  - Do not know
**5. What is your first language (Mother tongue)?**
  - English
  - French
  - Other (Please specify):
  - ________________________________
**6. What is your healthcare provider role (select all that are applicable)?**
  - Physician (please specify specialty if applicable: _______________________)
  - Physician Assistant
  - Nurse (please specify specialty if applicable: _______________________)
  - Trainee (please specify specialty if applicable: _______________________)
  - Admin coordinator
  - Care coordinator
  - Other (please specify): __________________
**7. Number of years working healthcare: _____________**
**8. Number of years in your current role: _____________**
